# Eco-Friendly Preparation of Silver Nanoparticles and Their Antiproliferative and Apoptosis-Inducing Ability against Lung Cancer

**DOI:** 10.3390/life12122123

**Published:** 2022-12-15

**Authors:** Vivekananthan Suseela, Ramalingam Nirmaladevi, Muthukrishnan Pallikondaperumal, Ramasamy Shanmuga Priya, Mohammed Rafi Shaik, Althaf Hussain Shaik, Mujeeb Khan, Baji Shaik

**Affiliations:** 1 Department of Biochemistry, P.S.G College of Arts and Science, Coimbatore 641014, Tamilnadu, India; 2 Department of Biochemistry, Biotechnology and Bioinformatics, Avinashilingam Institute for Home Science and Higher Education for Women, Coimbatore 641043, Tamilnadu, India; 3 Department of Microbiology, P.S.G College of Arts and Science, Coimbatore 641014, Tamilnadu, India; 4 Department of Chemistry, College of Science, King Saud University, P.O. Box 2454, Riyadh 11451, Saudi Arabia; 5 Department of Zoology, College of Science, King Saud University, P.O. Box 2454, Riyadh 11451, Saudi Arabia; 6 School of Chemical Engineering, Yeungnam University, Gyeongsan 38541, Republic of Korea

**Keywords:** anti-proliferative, apoptosis, green synthesis, silver nanoparticles, lung cancer

## Abstract

In the present study, the anti-proliferative and apoptotic potential of *Tabebuia roseo-alba* in lung cancer was assessed. Silver nanoparticles (AgNPs) of *T. roseo-alba* were synthesized using an ethanolic extract and characterized by adopting various parameters. Herein, the eco-friendly, cost-effective, and green synthesis of AgNPs was evaluated using an ethanolic extract of *T. roseo-alba*. The as-synthesized AgNPs were then characterized using various characterization techniques, such as UV-visible spectroscopy (UV-vis), X-ray powder diffraction (XRD), dynamic light scattering (DLS), scanning electron microscopy (SEM), and transmission electron microscopy (TEM). The AgNPs are crystalline, spherical, and highly stable AgNPs of varying sizes in the range of 5–20 nm. The anticancer activity of the ethanolic extract of *T. roseo-alba* and its AgNPs was determined using an MTT assay. The results indicated that, although both samples showed prominent anti-proliferative activity on lung cancer cell lines, the AgNPs of *T. roseo-alba* were found to be more potent than the ethanolic extract. Further, apoptosis induction ability was evaluated by FITC Annexin V and PI staining, the results of which demonstrated the efficiency of the ethanolic extract of *T. roseo-alba* and its AgNPs in causing oxidative stress and subsequent cellular death. This was subsequently further confirmed by measuring the mitochondrial membrane potential after staining the cells with JC1. The apoptotic mode of cell death was further confirmed by DNA fragmentation and caspase assays using Western blot analysis.

## 1. Introduction

Medicinal plants have been used to treat disease for centuries due to their therapeutic potential [1,2]. These plants produce a large number of diverse bioactive compounds, particularly secondary metabolites such as flavonoids, alkaloids, terpenoids, tannins, and phenolic compounds [3,4]. Researchers have become particularly interested in exploring these secondary metabolites to understand the mechanism of action of medicinal plants in the treatment of disease [5,6]. Recently, the time needed to isolate suitable phytocompound with drug-likeness and study its biological properties has been reduced owing to developments in the field of nanotechnology [7].

The use of nanoparticles (NPs) in the management of diseases is one of the aspects of nanomedicine, that is the application of nanotechnology in medicine [8,9]. Nanoparticles coated or coupled with various functionalities can be used to target the tumor sites, allowing for the early diagnosis of cancers and the elimination of tumors [10]. In hybrids of nanomaterials with pharmaceuticals, phytopharmaceuticals, etc., nanoparticles offer excellent flexibility in modifying fundamental properties of the resulting nanomedicines, including enhanced solubility, fast diffusivity, efficient drug release, and so on. In addition, nanomaterials-based medicines offer more effective routes of drug administration, increase product life, and lower therapeutic toxicity, etc., which ultimately reduce healthcare costs. Several noble metal NPs have been investigated for their anticancer potential, cellular imaging, and other oncological implications. Because of their enormous medical significance and potential to combat cancer, silver and its complexes have generated attention among the noble metals.

Although many chemical and physical methods are used to produce silver nanoparticles (AgNPs), biological methods are less hazardous, environmentally friendly, and more cost-effective [11,12]. Physical methods such as frame pyrolysis, ball milling, etc., are cheap and efficient for the large-scale production of nanomaterials, but they often generate low-quality, aggregated nanoparticles [13]. While chemical methods typically produce high-quality, controlled-size nanomaterials, they require hazardous chemicals and harsh reaction conditions. On the other hand, green methods are sustainable and potentially generate high-quality nanomaterial-based products with the minimum usage of hazardous substances [14]. Therefore, a battery of green approaches including sonochemical, electrochemical reduction methods and many more have been exploited for the preparation of different nanoparticles including Ag [15]. Moreover, the bioinspired methods which exploit the reduction properties of microorganisms, proteins, and secondary metabolites of plants have become popular in the last few decades [16]. Among these, plant extracts are preferred over other biosynthetic methods because they are readily available and include a diverse group of metabolites that favor the reduction of silver ions and accelerate the synthesis process [17,18,19]. Furthermore, the chemi-synthetic approach leaves harmful chemicals on the surfaces, which might be hazardous in medical settings [20].

Therefore, in the current study, the efficacy plants-based green method has been tested for the synthesis of biocompatible Ag NPs. To do this, *Tabebuiaroseo-alba* (Ridl) Sand (*T. roseo-alba)*, commonly known as the White Trump pet tree, has been used as both a reducing and stabilizing agent [4]. Gas chromatography–mass spectrometry (GC-MS) analysis of the ethanolic extract of *T. roseo-alba* leaves revealed the presence of diverse bioactive constituents which may be accountable for its various pharmacological activities. The leaves of this plant species are found to have strong free radical scavenging properties and powerful cytotoxic activity [21,22]. Therefore, we attempted to elucidate the therapeutic potential of *T. roseo-alba* and its AgNPs in lung cancer cell lines in vitro.

## 2. Materials and Methods

### 2.1. Collection and Preparation of Ethanolic Leaf Extract

Fresh leaves of *T. roseo-alba* were collected on the campus of the P.S.G College of Arts and Science, Coimbatore District, and authenticated at the Botanical Survey, Tamil Nadu Agriculture University (TNAU) Coimbatore, India (BSI/SRC/5/23/2019/Tech/3236). Fresh *T. roseo-alba* leaves were air-dried for a period of 2 to 3 weeks at room temperature, powdered, and extracted with ethanol using a Soxhlet extraction apparatus. The standard soxhlet extraction method was followed to obtain maximum yield with essential phytochemicals. The 500 g of fine powdered form of *T. roseo-alba* leaf sample was added individually in 2500 mL of ethanol (~99%) from Sigma-Aldrich (St. Louis, MO, USA). The sample containing solvent blends was separately heated at 70 °C for 1 h. After the extraction process, the solvent extract was filtered and then concentrated using a vacuum evaporator, which produces 39 g of powder. The collected residue was stored in airtight containers at 4°C until further use. To use the extract for the synthesis of Ag NPs, a stock solution was made by dissolving 1.0 g of *T. roseo-alba* extract in 10 mL of DI water.

### 2.2. Synthesis of Silver Nanoparticles

In a sterile conical flask, 90 mL of 1 mM silver nitrate solution was added to 10 mL of ethanolic plant extract. The solution was mixed thoroughly and continuously stirred using a magnetic stirrer at 800 rpm for 30 min [23]. The AgNPs formation was indicated by the change in the color of the solution from light yellow to dark brown which may be due to the reduction of silver ions by the ethanolic plant extract.

### 2.3. Characterization of Crude Extract and AgNPs of T. roseo-alba

Preliminary characterization was performed using UV-vis spectroscopy. The bio-reduction of Ag+ ions was examined by measuring the UV-vis spectra using 0.5 mL of the diluted aliquot with deionized water as a reference. UV-vis spectroscopic analysis of the biosynthesized AgNPs were carried out using a UV-vis spectrometer (Shimadzu Biospec Nano, Tokyo, Japan) wavelength of 300 to 800 nm. The presence and reduction of silver ions were indicated by a peak of absorption in the 300–500 nm range [24]. The functional groups of the phytochemicals involved in the bioreduction of Ag ions and subsequent capping and stabilization of biosynthesized AgNPs were determined using Fourier transform infrared (FTIR) analysis [25]. This was performed using a Shimadzu 8400S Fourier-transform infrared spectrometer operated at a resolution of 4 cm^−1^ in the range of 400–4000 cm^−1^. The size distribution and zeta potential of the bio-reduced AgNPs were assessed by dynamic light scattering (DLS) (Zetasizer Nano ZS, ZEN 3600, Malvern, UK). This equipment can measure particle size distributions in the range of 2–3 nm at 25 °C. X-ray diffraction spectroscopy, reveals the crystalline structure, size, and shape of the unit cell and the lattice parameters. The characterization of purified AgNO_3_ was carried out with an X-Pert Pro PANalytical diffractometer operating at a voltage of 40 kV and current of 30 mA with Cu Kα radiation in 2θ configurations. The average crystallite domain size D was calculated from the diffraction pattern using the Debye–Scherer formula [26]. TESCAN-MIRA3 XMU coupled with an energy-dispersive X-ray spectrometer was used to study the morphology and elemental analysis of THE biosynthesized AgNPs. The lyophilized AgNPs were sonicated using distilled water, and a drop coating of the nanoparticle suspension on glass slides was made and dried at ambient temperature. Energy dispersive X-ray spectroscopy (EDS) was used to perform a compositional analysis of the sample, and the presence of elemental silver was confirmed through EDS [27]. TEM was used to measure the size and shape of the AgNPs and to perform the morphological examination of the nanoparticles. The TEM micrograph images were captured on the JEOL 1200 EX instrument, operated at 100–200 kV. High-resolution images were captured and selected area electron diffraction (SAED) patterns were observed [28]. Plant extract GC-MS analysis was performed using a GC SHIMADZU QP2010 instrument and gas chromatograph interfaced to a mass spectrometer equipped with an RXi-1 ms fused silica capillary column (length: 30.0 m, diameter: 0.25 mm, film thickness: 0.25 μm composed of 100% dimethyl polysiloxane) to identify the existence of bioactive compounds extant in the plant extract [21]. Further details of the GC-MS analysis of ethanolic extract of *Tabebuia rosea-alba* leaves are provided in the Supplementary File (Figure S1, and Tables S1 and S2).

### 2.4. Measurement of Apoptosis

#### 2.4.1. MTT Dye Reduction Assay

Cell viability was examined by the reduction of 3-(4,5- dimethylthiazol-2- yl)-2,5 diphenyltetrazolium bromide (MTT) [29]. To do this, 96-well plates were used for the seeding of 1 × 105 cells/mL via incubation at 37 °C in 5% CO_2_ for one day. After this, the culture medium was changed, and different consecrations of the AgNPs were added and then incubated for one more day. Subsequently, the culture medium was taken out and washed with phosphate buffer saline (PBS). The cell viability of treated and untreated cells was measured using the absorbance values of formazan crystals used in the MTT reagent. Finally, the absorbance was measured at 560 nm using a microplate reader. The experiments were conducted in triplicate. Cell viability percentages were calculated according to the following equation [30]:

Cell viability (%) = Absorbance of experimental sample/Absorbance of experimental control × 100

#### 2.4.2. Analysis of Apoptosis by Annexin V/FITC Staining

The magnitude of apoptosis was determined using the ethanolic extract of *T. roseo-alba* and their AgNPs were determined using Annexin V/FITC Apoptosis Detection Kit (BD Biosciences, Haryana, India).

#### 2.4.3. Analysis of Mitochondrial Membrane Potential by JC-1 Staining

The apoptotic potential of the ethanolic extract of *T. roseo-alba* and its AgNPs was also determined using the JC-1 staining kit procedure.

#### 2.4.4. Analysis of DNA Fragmentation

Cells (1 × 10^6^ cells) were independently suspended in 10 mL of buffer containing 10 mM Tris-HCl and 10 mM EDTA (pH 8.0). The cells were treated with 10 mL of a solution containing 10 mM Tris-HCl, 10 mM EDTA (pH 8.0), 2% SDS, and 20 mg/mL proteinase K. The mixture was incubated at 37 °C for 3 h, followed by DNA extraction with phenol:chloroform: isoamyl alcohol solution (25:24:1). DNA was treated with DNase free RNase at a concentration of 20 mg/mL at 4 °C for 45 min and precipitated with 100 mL of 2.5 M sodium acetate and three volumes of ethanol. DNA fragmentation analysis was conducted with 100 base pairs of marker DNA and analyzed using electrophoresis for 1 h at 20 V on a 2% agarose gel containing ethidium bromide.

#### 2.4.5. Western Blotting

The regulation of apoptotic protein (caspase-3) in the treated cells was evaluated using Western blot analysis.

#### 2.4.6. Cell Cycle Analysis

The effect of the ethanolic extract of *T. roseo-alba* and its AgNPs on the cell cycle distribution of A549 cells was examined using flow cytometry.

## 3. Results and Discussion

### 3.1. UV-Visible Spectroscopy

Upon exposure to ethanolic plant extract, silver ions were reduced to AgNPs, wherein the change in color from light yellow to dark brown could be due to surface plasmon resonance excitation led by the collective oscillations of the valence electrons present in the electromagnetic field of incident radiation [31]. Thus, it is evident from the visual observations that the plant extract of *T. roseo-alba* was a good reducing agent for the reduction of Ag+ ions to AgNPs.

**Figure 1:**
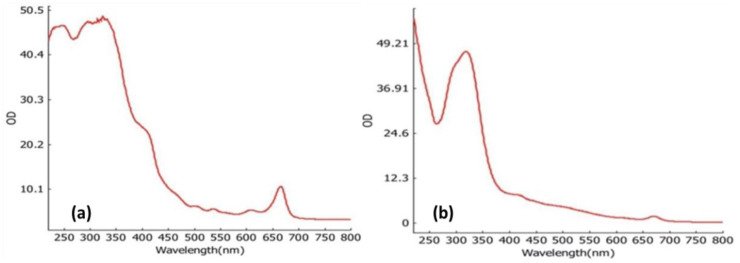
UV-vis analysis of (**a**) *Tabebuia roseo-alba* extract and (**b**) as-synthesized AgNPs.

The presence of various active metabolites in the extract was identified using UV-visible spectroscopy at wavelengths ranging from 220 to 800 nm. The absorption spectra of the ethanolic extract of the *T. roseo-alba* and its AgNPs are shown in Figure 1a and Figure 1b, respectively. Distinct peaks were observed at different wavelengths indicating the presence of various phytochemical constituents and their derivatives in the samples. In addition, the peak at 300 nm clearly confirmed the presence of phenolic compounds and their derivatives.

The reduction of silver ions by the plant extract and formation of AgNPs were progressively observed and measured with a UV-visible spectrometer at wavelengths in the range of 200–700 nm. UV-vis spectroscopy is a very helpful and practical approach for monitoring AgNP synthesis and stability, as well as primary characterization of the produced nanoparticles. The reduction of silver ions could be correlated with the respective UV-vis absorption spectrum which showed the surface plasmon resonance derived from the AgNPs around 400–450 nm. In addition, the number of absorption peaks and the breadth of SPR bands are related to the size distribution and shape of AgNPs in solution. The anisotropy of AgNPs was represented by several surface plasmon resonance (SPR) bands, whereas a single band primarily reflected spherical AgNPs. Furthermore, according to the quantum size theory, the SPR bands broaden as particle size decreases [32]. Usually, the optical absorption of metallic NPs is associated with SPR which may shift toward the blue or red end of the spectrum depending on the size, shape, aggregation of NPs, and the surrounding dielectric medium [33]. In most cases, a broad absorption band at higher wavelengths indicates a larger particle size. The position and width of the absorption band of colloidal silver particles typically depend on the density of free electrons, therefore an increase in the electron density of the particles results in the shifting of the absorption toward higher wavelengths [34]. The spherical shape of AgNPs was confirmed by the single SPR band recorded in our study. However, the UV spectrum of pure AgNPs obtained using *T. roseo-alba* extract exhibited two absorption bands, an intense band at ~330 nm, and a broad and weak absorption at ~430 nm as shown in Figure 1b. As the characteristic peak of Ag typically occurs between 400 and 450 nm, the peak at ~430 nm can be clearly attributed to the formation of AgNPs. To investigate the intense peak at ~330 nm, the UV spectrum of the pure *T. roseo-alba* extract was measured (Figure 1a). As expected, the UV spectrum of the plant extract exhibited an intense absorption band in the same region between 300 and 350 nm. Therefore, the appearance of this peak in AgNPs may be due to the presence of residual plant extract phytomolecules that remain on the surface of Ag NPs as stabilizing agents.

### 3.2. Fourier Transform Infrared Spectroscopy

FTIR is a highly accurate analytical technique for detecting and displaying components, chemical structures, functional groups, and molecular bonding configurations. Thus the characterization of the AgNPs using FTIR is used to ascertain the components that act as coating and stabilizing agents [35].

**Figure 2:**
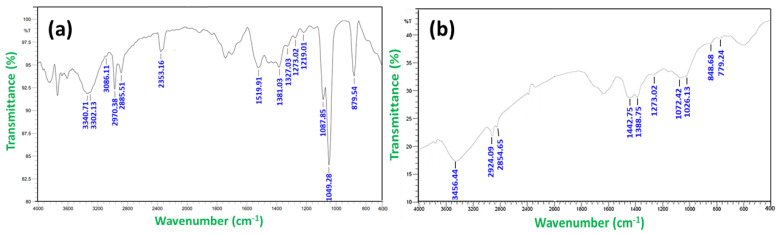
FT-IR spectrum of (**a**) *Tabebuia roseo-alba* and (**b**) as-synthesized AgNPs using *Tabebuia roseo-alba*.

FTIR analysis was performed to detect the bioactive components involved in the reduction of AgNO_3_ and confirm their efficient stabilization. The infrared (IR) spectra of the ethanolic extract of *Tabebuia roseo-alba* and its AgNPs are shown in Figure 2a and Figure 2b, respectively.

In the FT-IR spectrum of the ethanolic extract of *T. roseo-alba* the peaks at 3340.71 cm^−1^ and 3302.13 cm^−1^ corresponded to O-H vibration and stretching respectively, indicating the presence of the phenolic group. The peak at 2970.38 cm^−1^ corresponded to C-H stretching vibration which in turn indicates the presence of alkenes, while the peak at 1519.91 cm^−1^ corresponded to N-O asymmetric stretching vibration, which reveals the presence of nitro compounds. The peak at 1381.03 cm^−1^ was the result of N-O stretching vibration, which corresponded to nitro compounds, while peaks at 1273.02 cm^−1^ and 1219.01 cm^−1^ corresponded to C-O stretching vibration, demonstrating the existence of carboxylic acids, esters, ethers, and alcohols. Similarly, peaks at 1087.85 cm^−1^ and 1049.28 cm^−1^ corresponded to C-N stretching vibrations, revealing the presence of aliphatic amines. Finally, the peak at 879.54 cm^−1^ corresponded to N-H stretching vibration, revealing the presence of primary and secondary amines.

The FT-IR spectra of green synthesized AgNPs showed a shift in many peaks, which may be due to the involvement of phytochemicals in metallic nanoparticle synthesis. A vast shift at peak 3340.71 cm^−1^ to a higher wavelength was observed, suggesting that the hydroxyl groups of phenolic compounds play a considerable role in the capping of nanoparticles. The shift of peak at 2970.38 cm^−1^ to a lower wavelength implies that triterpenoids and saponins also play a role in nanoparticle synthesis [36].

The potential role of bioactive compounds such as flavonoids, phenols, alkaloids, and tannins in the extracts of *T. roseo-alba* favored the biosynthesis of nanoparticles. The absence of several basic groups and peaks of lower intensity can be seen in the IR spectra of AgNPs compared to the IR spectrum of the ethanolic extract which may be due to the involvement of the functional groups of the phytoconstituents in the leaf extract of *T. roseo-alba* in the reduction of silver ions.

### 3.3. Dynamic Light Scattering

DLS was used to estimate the size of the AgNPs by determining the diameter of the particles scattered in the colloidal suspension. DLS is a method for characterizing green synthesized AgNPs using phytoconstituents based on the concept of light scattering [37]. The polydispersity index (PDI) scale is dimensionless, wherein a value of “0” represents the maximum level of monodispersity and a value of “1” represents a polydisperse particle distribution [38].

**Figure 3:**
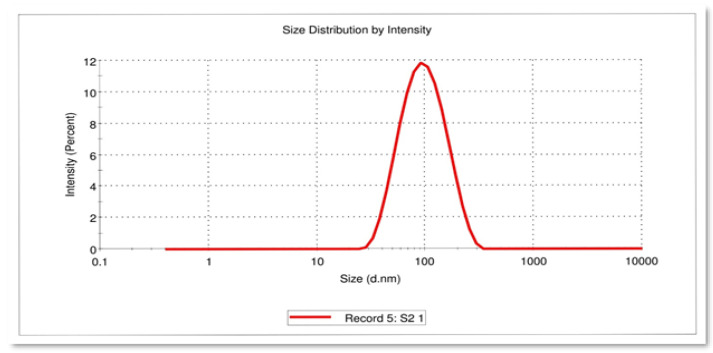
DLS image showing the size of the AgNPs synthesized using *T. roseo-alba*.

The DLS pattern of the present study revealed that the AgNPs synthesized from *T. roseo-alba* had a zeta average diameter in nanometers (d.nm) of 81.04 d.nm with a PDI of 0.224 (Figure 3). The size of the nanoparticle measured by DLS was considerably larger than that measured by TEM because the DLS method incorporates the hydrodynamic radius which includes the diameter of the particles and ions or molecules associated with the surface [39]. The hydration layer on the surface of AgNPs was included in the hydrodynamic size, which is often larger than that estimated from SEM images. The bioactive constituents present in the leaf extract may also play a role in the hydrodynamic size. For cell uptake, nanoparticle sizes of less than 150 nm and PDI values of less than 0.3 are sufficient [40]. Hence, the size and PDI value attained in the present study revealed the ability of green synthesized nanoparticles to target *T. roseo-alba* leaves to specific sites, extending the period of drug activity.

### 3.4. Zeta-Potential

The surface charge and stability of the nanoparticles were determined using zeta potential analysis. The velocity of the nanoparticles was measured under the influence of an electric field to assess their stability [41]. Colloidal dispersions are stable when the zeta potential falls within the range (larger than +30 mV and lesser than −30 mV) in the absence of steric stabilization [42].

**Figure 4:**
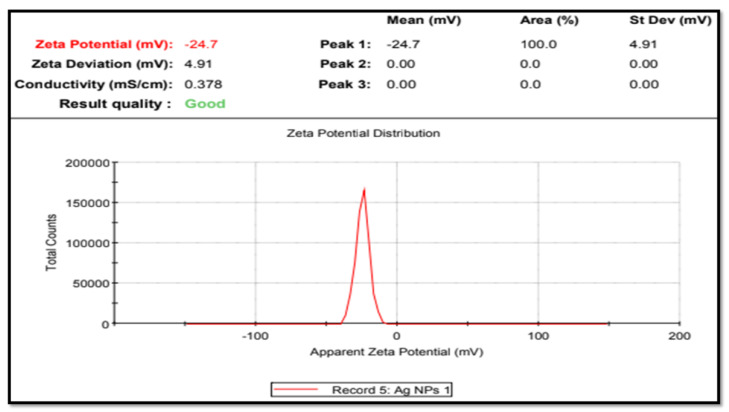
Zeta potential analysis of AgNPs synthesized using *T. roseo-alba*.

The zeta potential of the AgNPs of *T. roseo-alba* was found to be −24.7 mV (Figure 4). The negative potential value of AgNPs may be due to the coating of biofunctional constituents present in the plant extract. All suspended particles with a high negative or positive potential appeared to resist one another and have no tendency to flocculate, thereby promoting long-term stability, strong colloidal character, and high AgNPs dispersity.

The large negative or positive zeta potential of all suspended particles implies that they repel each other and that there is no propensity to flocculate, thus promoting long-term stability, strong colloidal character, and high AgNPs dispersity. On the other hand, a low negative or positive zeta potential indicates that the particles are prone to flocculation [43]. In the present study, the high negative zeta potential value indicates that there is repulsion among the AgNPs of *T. roseo-alba* confirming the stability of the particles. The high negative potential is associated with high stability, high colloidal quality, and good dispersion of AgNPs because of electrostatic repulsion [44].

### 3.5. X-ray Diffraction Analysis

**Figure 5:**
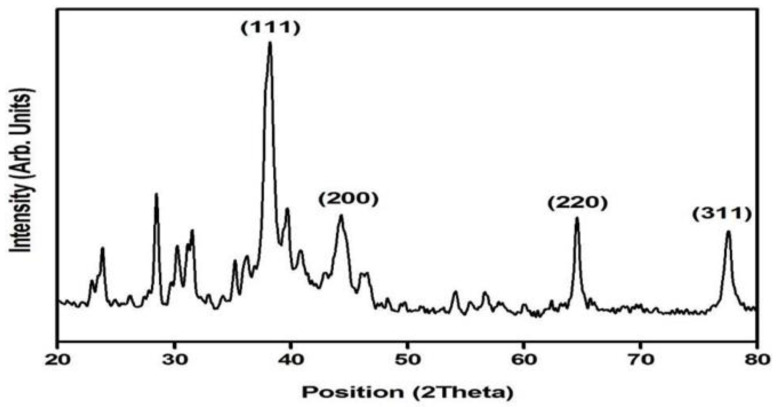
XRD analysis of AgNPs.

XRD is one of the most widely used techniques for characterizing NPs, enabling the collection of data on the crystalline structure, phase nature, lattice parameters, and crystalline grain size [45]. The phase formation of the biosynthesized AgNPs and the recorded X-ray diffraction pattern are shown in Figure 5. The diffraction peaks observed at the 2θ positions of 38.15°, 44.34°, 64.50°, and 77.48° confirm that Bragg reflections correspond to (111), (200), (220), and (311) planes, revealing the face-centered cubical phase formation of the prepared AgNPs (JCPDS card no; 03-065-2871).

The average crystallite size calculated with respect to the major intensity peak corresponds to the (111) plane and was found to be approximately 27 nm which is in good agreement with reports on the green synthesis of AgNPs. Along with the diffraction planes related to the cubical phase formation of AgNPs, a large number of minor reflections were also noted in the powder X-ray diffraction studies. A comparison of the phases of such impurity peaks revealed traces of AgNO_3_ which is the main ingredient used for the preparation of AgNPs. As the sample was prepared based on a green synthesis methodology using plant extract without any coarser draining of the sample with heat treatment, it would be difficult to completely eradicate the impurity phases. Moreover, in certain cases, inorganic residual contents of the plant extract remain on the surface of the Ag NPs, which is often reflected in the XRD spectrum of AgNPs [46]. Nevertheless, the presence of the characteristic peaks of Ag in the AgNPs diffractogram clearly indicates the formation of cubic AgNPs.

### 3.6. Energy Dispersive Spectroscopy

Energy dispersive spectroscopy (EDX), which involves X-rays interacting with the sample, was used to assess the elemental composition, relative abundance, and contaminants of the nanoparticles. The presence of silver crystallites was confirmed by the presence of emission peaks at 3 keV and the impurity-free nature of AgNPs was determined by observing the absence of any other peaks. The metabolite interaction with AgNPs on the surface was represented by the presence of other peaks such as carbon and oxygen [47].

**Figure 6:**
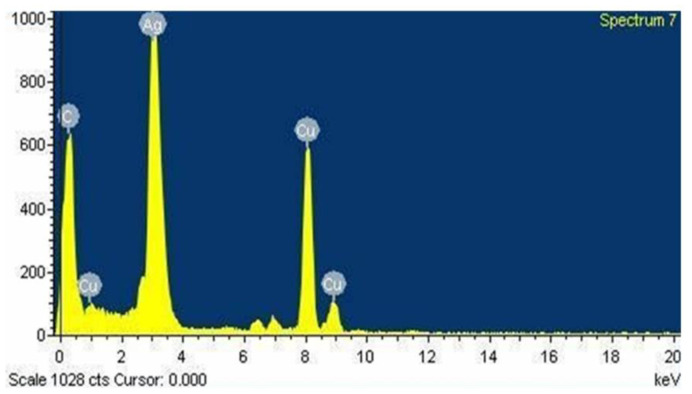
Energy dispersive X-ray spectrum of AgNPs synthesized using *Tabebuia roseo-alba*.

Figure 6 shows an EDX image of AgNPs synthesized from the ethanolic extract of *T. roseo-alba*. The results clearly indicate that the AgNPs of *T. roseo-alba* displayed an intense signal at 3 keV, which is characteristic of metallic AgNPs due to the relaxation of electronic states, which is reflected in the X-ray emission. The EDX analysis confirmed the weight percentage of silver as 48.34% obtained by using *T. roseo-alba*. Other weak signals with characteristic absorption for copper and carbon may be due to the presence of organic compounds present in the ethanolic plant extract, which represent the capping of photosynthesized AgNPs by the biomolecules present in the plant.

### 3.7. SEM Analysis

**Figure 7:**
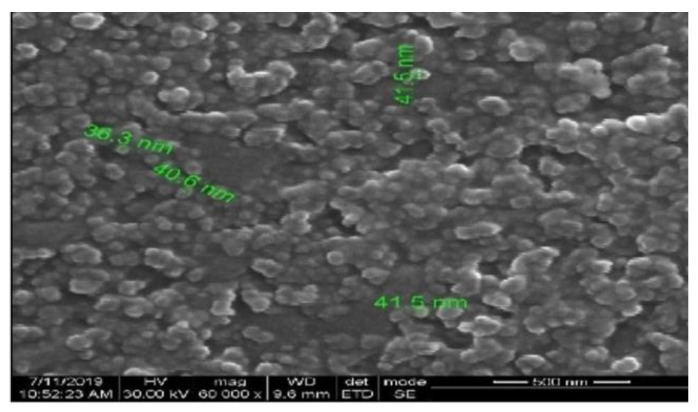
SEM image of AgNPs synthesized using *Tabebuia roseo-alba*.

SEM was used to obtain insights into the morphology and size of the AgNPs. From the data obtained from high-resolution images of the surface of nanoparticles, important attributes, including size, shape, topography, composition, and electrical conductivity can be studied [35]. The results of the study showed that spherical AgNPs were present in the sample, as shown in Figure 7.

### 3.8. Transmission Electron Microscope and Selected Area Electron Diffraction

TEM uses a beam of electrons to interact with an ultra-thin sample and provides the most precise and high-resolution imaging data on the morphology and dispersion of nanoparticles at nanometer resolution [45]. Selected area electron diffraction (SAED) is a technique performed along with TEM for the characterization of the crystallinity, and lattice parameters of nanoparticles and validates the XRD results [48].

**Figure 8:**
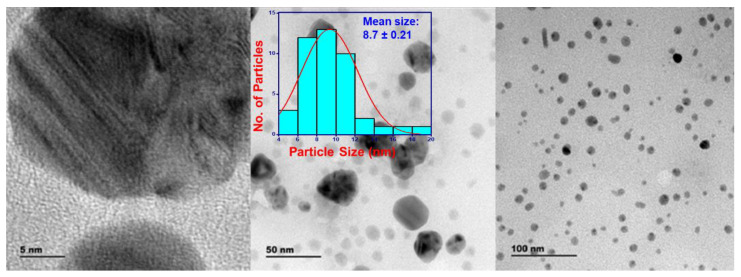
TEM image of AgNPs synthesized using *Tabebuia roseo-alba* captured at different resolutions.

The TEM images of the AgNPs synthesized using *T. roseo-alba* captured at 5, 50, and 100 nm scales are shown in Figure 8. The TEM micrographs of the green-synthesized AgNPs revealed that they were spherical in nature and it also suggested that the size of the particles were ranging between 5 and 20 nm.

**Figure 9:**
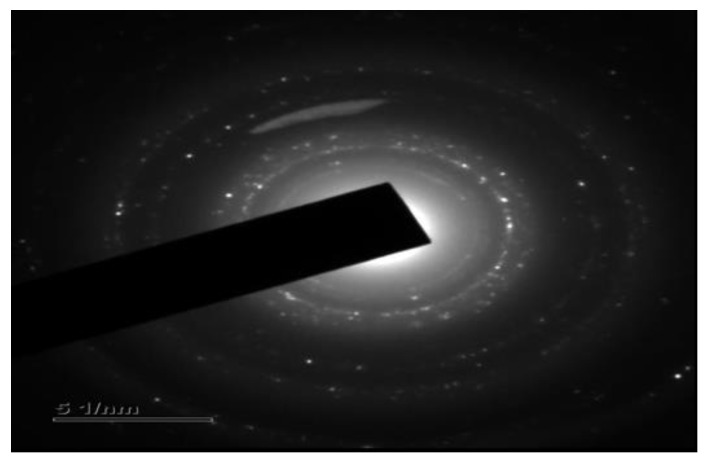
Selected area electron diffraction pattern of elemental silver.

The clear lattice fringes in the high-resolution TEM images and the typical SAED pattern (Figure 9), with bright circular rings corresponding to different lattice planes, revealed the highly crystalline nature of the sample. As shown in Figure 9, the SAED spots indicate different crystallographic planes of elemental silver’s face-centered cubic *(fcc)* structure.

From the results of TEM analysis, the formation of spherical, crystalline, and polydispersed AgNPs of varying sizes 5–20 nm were observed.

### 3.9. Tentative Mechanism of the T. roseo-alba-Mediated Formation of AgNPs

T. roseo-alba extract functioned as both a reducing and stabilizing agent to generate AgNPs. The corresponding process may involve three different stages: nucleation, growth, and termination [49,50]. During the induction phase (nucleation), the bio-reductant phytomolecules of *T. roseo-alba* facilitated the reduction of Ag ions to generate Ag seeds. These seeds continue to grow into larger aggregates during the growth process. Finally, in the termination phase, the resultant AgNPs were stabilized by the phytoconstituents of *T. roseo-alba* which dictated the final spherical morphology of nanoparticles. The extract of *T. roseo-alba* is known to contain a rich quantity of polyphenols, flavonoids, triterpenoids, and saponins etc., which have excellent antioxidant properties and thus may be involved in the reduction of the metal precursor.

## 4. Evaluation of Anticancer Potential and In Vitro Cytotoxicity of Ethanolic Extract of *T. roseo-alba* and Its Biosynthesized AgNPs

### 4.1. Cytotoxic Effect of the Ethanolic Extract and AgNPs of T. roseo-alba by MTT Assay

The MTT assay is a quantitative assay used to measure the cell viability and cell propagation. MTT is cleaved by the mitochondrial enzyme dehydrogenase in living cells to produce the purple formazan, which can be measured. Formazan product formation by live cells can be directly correlated with the number of viable cells and the extent of cytotoxicity [51].

**Figure 10:**
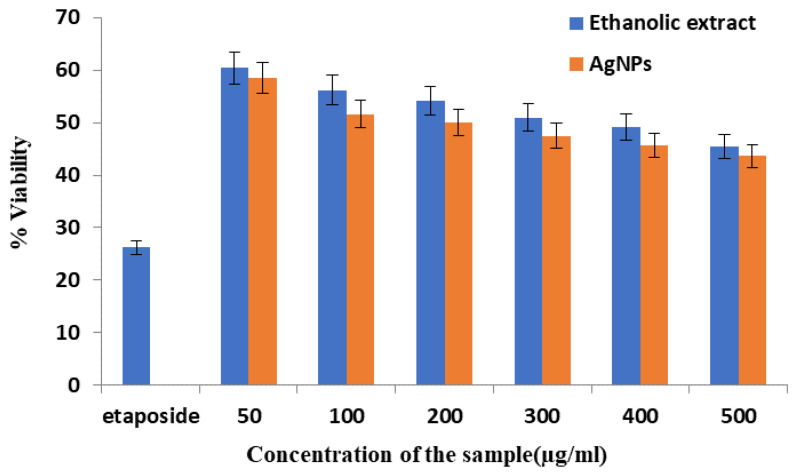
Cytotoxic effect of the ethanolic extract of *T. roseo-alba* and its AgNPs by MTT assay in terms of cell viability.

The cytotoxic effect of the ethanolic extract of *T. roseo-alba* and its AgNPs in lung cancer cells was assessed using an MTT assay. The experiment was performed using different concentrations of the ethanolic extract of *T. roseo-alba* and AgNPs (50, 100, 200, 300, 400, and 500 μg). The effect of the samples on cell viability is shown in Figure 10, with cytotoxicity recorded as IC_50_ (μg/mL).

These results indicate that the ethanolic extract of *T. roseo-alba* and its AgNPs induced cell death in a concentration-dependent manner. When A549 cells were treated with the samples, the viability of the cells decreased, even at the lowest concentration. Although both the samples showed promising cytotoxic activity, AgNPs of *T. roseo-alba* were relatively more potent than the ethanolic extract. Figure 10 demonstrates the comparative cytotoxic activity of both pure ethanolic extract of *T. roseo-alba* and as-prepared AgNPs in terms of cell viability. After the treatment with the samples, the surviving ability of cancer cells was slightly better in the case of pure extract when compared to as-prepared AgNPs, this is clearly reflected by the higher percentage of cell viability recorded after the treatment of cells with pure extract as shown in Figure 10. This behavior indicates that the anticancer property of AgNPs is superior when compared to the pure extract, as a greater number of cancer cells were destroyed by the treatment of AgNPs (thus less cell viability as shown in Figure 10). Further, greater cytotoxic effect was observed at their higher concentration (500 μg). The IC_50_ values were 300 μg/mL and 200 μg/mL for the ethanolic extract of *T*. *roseo-alba* and its AgNPs respectively. The dimensions of the NPs, amount of Ag, and coating agents contribute to greater cytotoxicity and decreased cell growth. Owing to their increased cellular absorption and wide surface area for interaction with biomolecules, small NPs were found to be more cytotoxic [20]. The reduction in cell viability with increasing concentrations of the ethanolic extract of *T. roseo-alba* and its AgNPs highlighted the ability of the plant to act as an effective drug for the treatment of cancer which could be attributed to the antioxidant and radical scavenging potential of the plant.

The cytotoxic effect of *T. roseo-alba* and its AgNPs may be ascribed to the presence of a high content of bioactive metabolites, such as phenols and flavonoids, which may be responsible for the formation of green synthesized AgNPs. These principal bioactive metabolites may have anti-neoplastic potential and the ability to cause cell death through molecular processes such as the activation of autophagy, the formation of ROS, and apoptosis.

### 4.2. Determination of Apoptosis

#### 4.2.1. Analysis of Apoptosis by Annexin V/FITC Staining

Apoptosis plays a crucial role in homeostasis and it is characterized by numerous morphological and biochemical alterations in the cells [52]. To determine apoptosis in lung cancer cells treated with IC_50_ concentrations of ethanolic extract *T. roseo-alba* and its AgNPs, FITC Annexin V and PI staining were performed, followed by flow cytometry analysis. Under normal conditions, phosphatidyl serine (PS) residues are present in the inner cytoplasmic membrane of cells. Phosphatidyl inositol serine is translocated to the cell membrane during the early stages of apoptosis and can be stained with fluorescently labeled annexin V [53].

**Figure 11:**
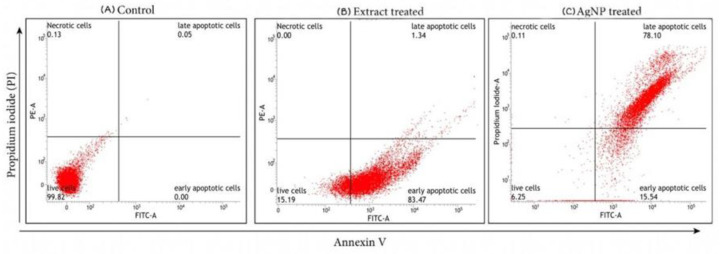
Flow cytometry analysis of A549 cells by double-labelling with Annexin V and PI dyes, (**A**) control, (**B**) ethanol extract of *Tabebuia roseo-alba,* (**C**) AgNPs of *T. roseo-alba*.

These results demonstrated the induction of apoptosis in cancer cells exposed to the ethanolic extract (300 μg/mL) and AgNPs (200 μg/mL) for 24 h. The ethanolic extract and AgNPs induced cell death by Annexin V/FITC staining were studied by flow cytometry in the lung cancer cell line A549, as shown in Figure 11.

After treatment with *T. roseo-alba* and its AgNPs, the apoptotic and non-apoptotic cell populations were evaluated by Annexin V/PI staining. The initiation of apoptosis was evident from the higher population of dead cells upon treatment. When the cells were treated with ethanolic extract of *T. roseo-alba*, they were observed in an early apoptotic stage of cell death indicating the ability of the ethanolic extract to induce apoptosis. Upon further treatment with its AgNPs, the cells were observed in late apoptosis highlighting the ability of AgNPs to induce apoptosis, with very few cells observed in early apoptosis. Since the AgNPs of *T. roseo-alba* has directed the cells into late apoptosis it can be concluded that the synthesized AgNPs have the ability to induce apoptosis.

#### 4.2.2. Analysis of Mitochondrial Membrane Potential by JC-1 Staining

Apoptosis is triggered when cells are destroyed by the development of reactive oxygen species in their internal environment during metabolic activity. Excessive reactive oxygen species (ROS) generation causes alterations in the mitochondrial membrane permeability, which harms the respiratory chain and causes cellular death. Mitochondria play an important role in cellular death, and alteration in the membrane permeability of mitochondria is one of the first steps in apoptosis. Numerous pro-apoptotic proteins are released into the cytoplasm by the mitochondria, and a permeability transition pore is formed in the mitochondrial membrane. Any alterations in the permeability of the mitochondrial membrane were detected by JC-1 staining. Because of the electrochemical potential gradient, JC-1 accumulates in the mitochondrial matrix and forms J-aggregates and red fluorescent aggregates, in normal cells. JC-1 dye clustering in the mitochondria is prevented by deviations in the mitochondrial membrane potential and hence, it is distributed throughout the cell. This results in a change in fluorescence from red (J-aggregates) to green (JC-1 monomers), indicating the depolarization of the mitochondrial membrane [54].

**Figure 12:**
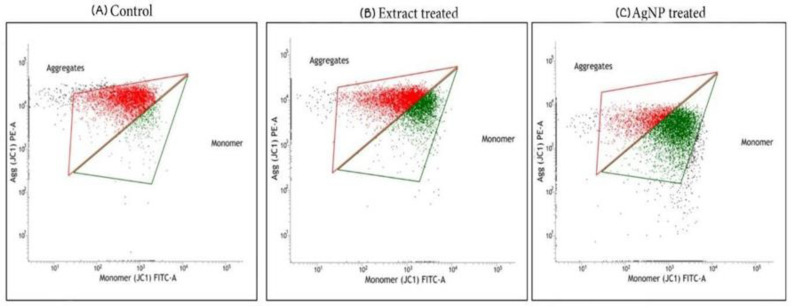
Flow cytometric analysis of A549 cell lines after staining with JC1, (**A**) control, (**B**) ethanol extract of *T. roseo-alba,* (**C**) AgNPs of *T. roseo-alba*.

In the present study, mitochondrial membrane potential was investigated using a flow cytometer after JC-1 staining. The proportion of cancer cells undergoing apoptosis was determined after treatment with the ethanolic extract of *T. roseo-alba* and AgNPs at their respective IC_50_ concentrations. The results are reported based on the red to green fluorescence ratio, where an increased ratio indicated the viability of the cells and a lesser ratio indicates cell death. The control revealed higher red to green fluorescence ratio, revealing fully polarized mitochondria, which formed J-aggregates as red fluorescence. Treatment with ethanolic extract and their AgNPs, on the other hand, caused mitochondrial membrane depolarization in A549 cells, as evidenced by a decrease in red fluorescence and an increase in green fluorescence intensity, indicating the ability of the *T. roseo-alba* samples to trigger apoptosis (Figure 12).

Compared to the ethanolic extract, increased green fluorescence intensity was observed upon exposure to AgNPs. This change in fluorescence pattern indicated the more pronounced ability of AgNPs to depolarize mitochondrial membrane integrity leading to apoptosis induction and subsequent cell death. The depletion of the mitochondrial membrane potential causes cytochrome C to be released into the cytoplasm and activates the upregulation of caspase-3 via the caspase-9 pathway [55]. These findings reiterate the ability of the ethanolic extract of *T. roseo-alba* and its AgNPs to activate the intrinsic apoptotic signaling pathway. This may be attributed to the generation of intracellular ROS and the depletion of mitochondrial membrane integrity, which ultimately results in the fragmentation of DNA, thereby causing cell cycle arrest, demonstrating the anti-proliferative activity of the ethanolic extract of *T. roseo-alba* and its AgNPs.

These results indicate that treatment with the ethanolic extract and AgNPs of *T. roseo-alba*, resulted in the depolarization of the mitochondrial membrane potential, and cytochrome C release, thereby triggering cells to undergo apoptosis through an intrinsic pathway. The results of cell line analysis clearly highlight the presence of active compounds in the extract, which could mediate the scavenging action of the radicals generated and coordinate nanoparticle formation. This in turn could readily affect the membrane stability of the mitochondria and initiate programmed cell death and interrupt cell cycle events proving its anti-proliferative action in cancer cells. Ultimately, this could aid in the design of drugs against oxidative stress-induced diseases, such as cancer.

#### 4.2.3. Analysis of DNA Fragmentation

**Figure 13:**
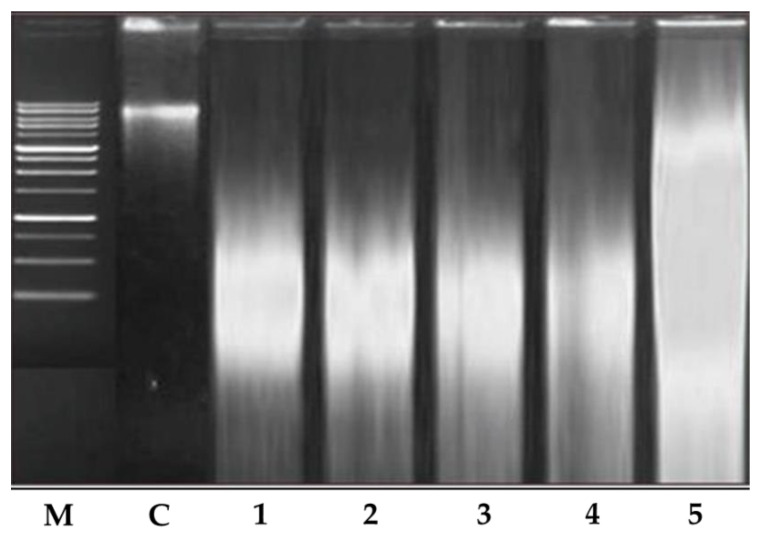
DNA fragmentation by *Tabebuiaroseo-alba* and its AgNPs. M—ladder marker. 1—200 µg/mL treated with ethanolic extract of *Tabebuia roseo-alba*. 2—400 µg/mL treated with ethanolic extract of *T. roseo-alba*. 3—500 µg/mL treated with ethanolic extract of *T. roseo-alba*. 4—400 µg/mL treated with AgNPs of *T. roseo-alba*. 5—500 µg/mL treated with AgNPs of *T. roseo-alba*.

To obtain further insights into the mechanism of cell death caused by the ethanolic extract of *T. roseo-alba* and its AgNPs, its effect on DNA fragmentation was determined. Induction of apoptosis in A549 cells was validated by DNA fragmentation analysis using gel electrophoresis, as evidenced by a dose-dependent increase in DNA fragmentation upon treatment. The banding pattern observed (Figure 13) indicated the occurrence of DNA shearing and fragmentation in the lanes treated with increasing concentrations of *T. roseo-alba* and its AgNPs indicative of apoptosis induction.

### 4.3. Western Blotting

ROS were generated by an ethanolic extract of *T. roseo-alba* and its AgNPs, which inhibited the cell cycle at specific checkpoints in A549 cells, resulting in self-mediated cellular death or DNA repair. Variations in the expression of certain protein cause cellular apoptosis via the mitochondrial pathway. For example, caspases are known to play a key role in apoptosis execution and initiation, and are stimulated in many cells throughout this process. Activated caspase-3 plays a crucial role in genomic fragmentation and programmed cell death is crucial. Furthermore, caspase 3 has been shown to cleave and translocate caspase-activated DNAse (CAD) upon activation, resulting in DNA fragmentation [52].

Variations in the Bax/Bcl ratio cause outer mitochondrial membrane permeabilization, the release of soluble proteins from the inter membrane gap into the cytosol, and enhanced caspase activation. Effector caspase (caspase-3) is activated by caspase-9, and the resulting activated caspase 3 cleaves substrates at aspartate residues. This appears to be a prominent apoptotic incident [54].

**Figure 14:**
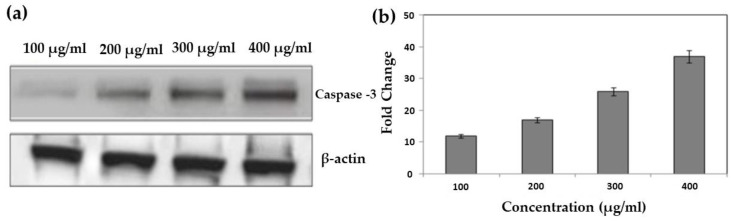
Western blotting for caspase 3 expression on human lung cancer A549 cells treated with ethanolic extract of *Tabebuia roseo-alba;* (**a**) protein abundance of active caspase-3 and control β-actin, and (**b**) densitometry for the protein abundance of active caspase 3.

**Figure 15:**
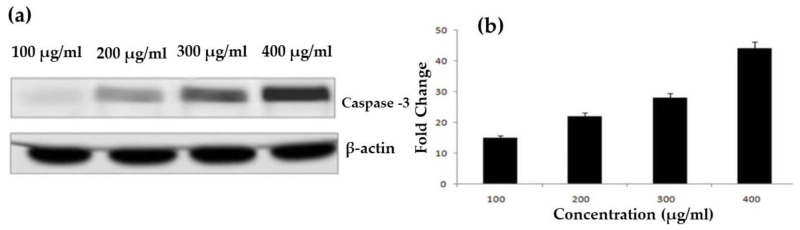
Western blotting for caspase 3 expression on human lung cancer A549 cells treated with AgNPs of *Tabebuia roseo-alba;* (**a**) protein abundance of active caspase 3 and control β-actin, and (**b**) densitometry for the protein abundance of active caspase 3.

To determine the effect of the ethanolic extract of *T. roseo-alba* and its AgNPs on the apoptotic pathway, caspase-3 activity was examined in A549 cells using Western blotting. As predicted, upon treatment with ethanolic extract of *T. roseo-alba* and its AgNPs, the level of caspase 3 expression was observed to be concentration-dependent. The treatment of *T. roseo-alba*, with the ethanolic extract, triggered apoptosis in A549 cells, resulting in an up-regulation of the active forms of caspases 3. Further, when A549 cells were treated with AgNPs of *T. roseo-alba*, more pronounced expression levels of caspases-3 were observed compared to the ethanolic extract of *T. roseo-alba* (Figure 14 and Figure 15).

These results indicate that treatment with the ethanolic extract of *T. roseo-alba* and its AgNPs mediated apoptosis by intrinsic pathway. The change in membrane potential resulted in the development of a transition pore in the mitochondria, which led to the release of cytochrome C. In turn, this initiated the caspase cascade resulting in DNA fragmentation.

### 4.4. Cell Cycle Analysis

**Figure 16:**
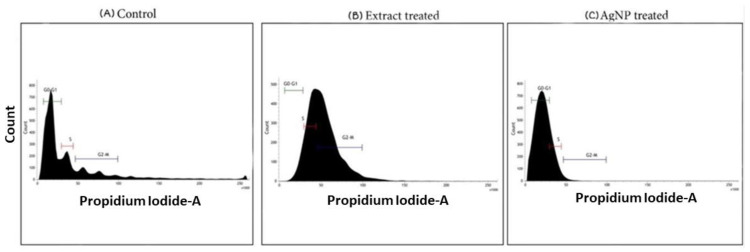
Flow cytometric analysis of cell cycle distribution of A549 cells.

Apoptosis and cell cycle arrest are inextricably linked because apoptosis occurs when cell proliferation is disrupted at specific cell cycle checkpoints. When DNA damage occurs in cells, a checkpoint in each step of the cell cycle is responsible for stopping cell growth until the damage is repaired, or if the damage cannot be repaired, cells undergo apoptosis [56]. The effect of the ethanolic extract of *T. roseo-alba* and its AgNPs on the cell cycle distribution of A549 cells was examined using flow cytometry. As depicted in Figure 16, cells treated with ethanolic extract at their IC_50_ were arrested at the G2/M transition stage, as evidenced by the increased percentage of the cell population. This demonstrates the ability of ethanolic extract-treated A549 cells to pass through the G2 checkpoint, thereby indicating that the G2/M transition was disrupted upon treatment with the ethanolic extract.

Further treatment with AgNPs blocked the cell cycle at the G0-G1 checkpoint, at which point the cells were found to be quiescent. In contrast, the untreated control cells were distributed in all the phases of the cell cycle and the results further support the more pronounced apoptosis-inducing ability of AgNPs of *T. roseo-alba* compared to the ethanolic extract.

Cell cycle analysis performed using the samples of *T. roseo-alba* revealed that the A549 cells were able to undergo cell cycle arrest proving their antiproliferative effect. Cells treated with *T. roseo-alba* showed cell cycle phase transition and were arrested at the G2/M phase, whereas the green synthesized nanoparticles were arrested at the G0/G1 phase indicating the prominent effect of nanoparticles when compared to the crude extract. These results highlighted the ability of AgNPs to control cancer cell proliferation by blocking the cell cycle at specific checkpoints that suppress cancer progression.

## 5. Conclusions

In the present study, eco-friendly, cost-effective green synthesis of AgNPs was explored. A reduction in silver ions by the bioactive constituents found in the ethanolic extract of *T. roseo-alba* was confirmed by the UV absorption peak between 400 and 450 nm. The involvement of various functional groups in the formation and stabilization of AgNPs was further validated by using FTIR analysis. The crystalline nature of the AgNPs was confirmed based on the XRD pattern and the high negative potential observed in the DLS analysis ensured an adequate repulsion among the particles and greater stability of the green synthesized nanoparticles. Morphological assessment using SEM and TEM revealed a size distribution of the particles in the range between 5 and 20 nm. The MTT cell viability assay results indicated that both the ethanolic extract of *T. roseo-alba* and its AgNPs induced toxicity in lung cancer cells in a concentration-dependent manner. The apoptosis-inducing ability and antiproliferative potentials of *T. roseo-alba* leaves were ascribed to the major bioactive molecules, such as polyphenols and flavonoids present in them. The ethanolic extract of *T. roseo-alba* and its AgNPs also increased the level of intracellular ROS and amplified the autophagy and apoptosis rate by inducing membrane permeability, and the depolarization of the mitochondrial membrane potential which was evident from the results of Annexin V/FITC and JC-1 staining. Furthermore, cell cycle analysis using the ethanolic extract of *T. roseo-alba* and its AgNPs resulted in the build-up of cells in the G2/M and G0-G1 phases, respectively, indicating apoptotic cell death. Based on these results, it may be concluded that AgNPs of *T. roseo-alba* leaf extract showed more pronounced apoptotic induction and antiproliferative activity than the ethanolic extract of *T. roseo-alba* and may thus be considered a potential anticancer drug for use in the treatment of lung cancer.

## Data Availability

Data contained within the Article.

## Supplementary Materials

The following supporting information can be downloaded at: https://www.mdpi.com/article/10.3390/life12122123/s1. Figure S1: GC-MS analysis of ethanolic extract of *Tabebuia rosea-alba* leaves. Table S1: Bioactive constituents identified in the ethanolic extract of *Tabebuia rosea-alba* leaves by GC-MS analysis. Table S2: Biological activities of the phytoconstituents identified in the ethanolic extract of *Tabebuia rosea-alba* leaves by GC-MS analysis.
