# Supplementary material for: Eco-Friendly Preparation of Silver Nanoparticles and Their Antiproliferative and Apoptosis-Inducing Ability against Lung Cancer
Source: Life (Basel). 2022 Dec 15;12(12):2123. doi: 10.3390/life12122123 (PMC9782107; doi:10.3390/life12122123)
Supplement: Supplementary file 1 [file life-12-02123-s001.zip › life-2080086-supplementary.pdf]

life

MDPI

Supplementary File

Eco-Friendly Preparation of Silver Nanoparticles and Their Antiproliferative and Apoptosis-Inducing Ability against Lung Cancer

Vivekananthan Suseela [1,*], Ramalingam Nirmaladevi [2], Muthukrishnan Pallikondaperumal [3], Ramasamy Shanmuga Priya [3], Mohammed Rafi Shaik [4], Althaf Hussain Shaik [5], Mujeeb Khan [4] and Baji Shaik [6,*]

1 Department of Biochemistry, P.S.G College of Arts and Science, Coimbatore 641014, Tamilnadu, India
2 Department of Biochemistry, Biotechnology and Bioinformatics, Avinashilingam Institute for Home Science and Higher Education for Women, Coimbatore 641043, Tamilnadu, India; nirmaladevisaravanan32@gmail.com
3 Department of Microbiology, P.S.G College of Arts and Science, Coimbatore 641014, Tamilnadu, India; pmamatricschool@gmail.com (M.P.); priyajasper@gmail.com (R.S.P.)
4 Department of Chemistry, College of Science, King Saud University, P.O. Box 2454, Riyadh 11451, Saudi Arabia; mrshaik@ksu.edu.sa (M.R.S.); kmujeeb@ksu.edu.sa (M.K.)
5 Department of Zoology, College of Science, King Saud University, P.O. Box 2454, Riyadh 11451, Saudi Arabia; salthaf@ksu.edu.sa
6 School of Chemical Engineering, Yeungnam University, Gyeongsan 38541, Republic of Korea
* Correspondence: suseelavivek@gmail.com (V.S.); shaikbaji@yu.ac.kr (B.S.)

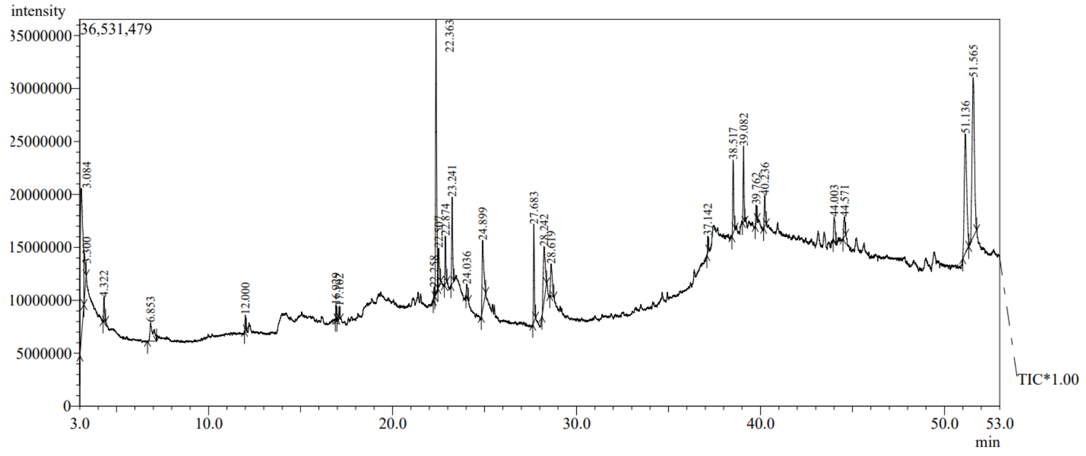

Figure S1: GC-MS analysis of ethanolic extract of *Tabebuia rosea-alba* leaves [1].

Table S1: Bioactive Constituents Identified in the Ethanolic Extract of *Tabebuia rosea- alba* Leaves by GC-MS Analysis [1].

RT	Molecular formula	Molecular weight	Name of the compound
22.367	$C_{20}H_{40}O$	296	2-hexadecen-1-ol<br>3,7,11,15,-tetramethylhexadec-2-en-1-ol<br>3,7,11,15-tetramethyl-2-hexadecen-1-ol
22.508	$C_{20}H_{40}$	280	2-hexadecene<br>3,7,11,15-tetramethyl-[R-[R*R*-(E)]<br>3,7,11,15-tetramethyl-2-hexadecene
24.900	$C_{18}H_{34}O_2$	282	9-octadecenoic acid (Z)<br>octadec-9-enoic acid
27.683	$C_{20}H_{40}O$	296	Phytol<br>2-hexadecen-1-ol<br>trans – Phytol<br>3,7,11,15-tetra methyl-2-hexadecen-1-ol
28.242	$C_{20}H_{34}O_2$	306	8,11,14- eicosatrienoic acid (Z,Z,Z)

39.083	$C_{30}H_{50}$	410	Squalene<br>2,6,10,14,18,22-tetracosahexaene<br>Spinacene
51.136	$C_{29}H_{50}O$	414	Stigmast-5-en-3-ol<br>(3.BETA.,24S) stigmast-5-en-3-ol<br>Beta-Sitosterol<br>Stigmast-5-en beta-ol<br>Alpha-dihydrofucosterol
51.567	$C_{29}H_{50}O$	414	Stigmast-5-en-3-ol<br>(3.BETA.,24S) stigmast-5-en-3-ol

Table S2: Biological Activities of the Phytoconstituents Identified in the Ethanolic Extract of *Tabebuia rosea-alba* Leaves by GC-MS Analysis [1].

% Area	Name of the compound	Biological activity**
12.57	2-hexadecen-1-ol<br>3,7,11,15,-tetramethylhexadec-2-en-1-ol<br>3,7,11,15-tetramethyl-2-hexadecen-1-ol	Antimicrobial, Anticancer,<br>Anti-inflammatory, Diuretic
2.01	2-hexadecene<br>3,7,11,15-tetramethyl-[R-[R*R*-(E)]<br>3,7,11,15-tetramethyl-2-hexadecene	Transplant rejection treatment,<br>Anticarcinogenic,<br>Multiple sclerosis treatment,<br>Gaucher disease treatment,<br>Metabolic disease treatment.
5.18	9-octadecenoic acid (Z)<br>octadec-9-enoic acid	Cancer preventive,<br>Flavor, Hypocholesterolemic,<br>5-Alpha reductase inhibitor,<br>Antiandrogenic, Perfumery, Insectifuge,<br>Anti-inflammatory, Anemiagenic,<br>Dermatitigenic, Choleretic.
3.54	Phytol<br>2-hexadecen-1-ol<br>trans – Phytol<br>3,7,11,15-tetra methyl-2-hexadecen-1-ol	Pesticide, Perfumery, Anti-feedent,<br>Haepato protective, Anti-inflammatory,<br>Analgesic, Antibacterial activity.
4.80	8,11,14- eicosatrienoic acid (Z,Z,Z)	Cardio protective, Hypocholesterolemic,<br>Anticancer, Anticoronary.
2.85	Squalene<br>2,6,10,14,18,22-tetracosahexaene<br>Spinacene	Antibacterial, Antitumor, Antioxidant,<br>Immunostimulant, Hypocholesterolemic,<br>Lipoxygenase-inhibitor.
12.42 and 16.21	Stigmast-5-en-3-ol<br>(3.BETA.,24S) stigmast-5-en-3-ol<br>Beta-Sitosterol<br>Stigmast-5-en beta-ol<br>Alpha-dihydrofucosterol	Cytotoxicity against human, Diuretic,<br>Hepatoprotective, Anti-microbial, Anti-inflammatory, Anticancer,<br>Antiasthma.

**Source: Dr. Duke's phytochemical and ethnobotanical databases (Online database).

Reference:

1. Suseela, V.; Nirmaladevi, R. Quantitative Phytochemical Screening and GC-MS Analysis of the Ethanolic Extract of *Tabebuia roseo-alba* (*Ridl*) Sand. *Indian J. Nutr. Diet.* **2021**, *58*, 317–325.
